# Neuropeptidomic analysis of the embryonic Japanese quail diencephalon

**DOI:** 10.1186/1471-213X-10-30

**Published:** 2010-03-18

**Authors:** Birger Scholz, Henrik Alm, Anna Mattsson, Anna Nilsson, Kim Kultima, Mikhail M Savitski, Maria Fälth, Karl Sköld, Björn Brunström, Per E Andren, Lennart Dencker

**Affiliations:** 1 Department of Pharmaceutical Biosciences, division of toxicology, Uppsala University, The Biomedical Center, Husargatan 3, Box 594, SE-75124 Uppsala, Sweden; 2 Department of Pharmaceutical Biosciences, Medical Mass Spectrometry, Uppsala University, The Biomedical Center, Husargatan 3, Box 583, SE-75123 Uppsala, Sweden; 3 Department of Environmental Toxicology, Uppsala University, Norbyvägen 18A, SE-75236 Uppsala, Sweden; 4 Department of Cellular and Molecular Biology, Uppsala University, The Biomedical Center, Husargatan 3, Box 596, SE-75124 Uppsala, Sweden; 5 Denator AB (since 2006), Uppsala Science Park, Dag Hammarskjolds road 32A, SE 751 83 Uppsala, Sweden; 6 Centre for Reproductive Biology in Uppsala

## Abstract

**Background:**

Endogenous peptides such as neuropeptides are involved in numerous biological processes in the fully developed brain but very little is known about their role in brain development. Japanese quail is a commonly used bird model for studying sexual dimorphic brain development, especially adult male copulatory behavior in relation to manipulations of the embryonic endocrine system. This study uses a label-free liquid chromatography mass spectrometry approach to analyze the influence of age (embryonic days 12 vs 17), sex and embryonic day 3 ethinylestradiol exposure on the expression of multiple endogenous peptides in the developing diencephalon.

**Results:**

We identified a total of 65 peptides whereof 38 were sufficiently present in all groups for statistical analysis. Age was the most defining variable in the data and sex had the least impact. Most identified peptides were more highly expressed in embryonic day 17. The top candidates for EE_2 _exposure and sex effects were neuropeptide K (downregulated by EE_2 _in males and females), gastrin-releasing peptide (more highly expressed in control and EE_2 _exposed males) and gonadotropin-inhibiting hormone related protein 2 (more highly expressed in control males and displaying interaction effects between age and sex). We also report a new potential secretogranin-2 derived neuropeptide and previously unknown phosphorylations in the C-terminal flanking protachykinin 1 neuropeptide.

**Conclusions:**

This study is the first larger study on endogenous peptides in the developing brain and implies a previously unknown role for a number of neuropeptides in middle to late avian embryogenesis. It demonstrates the power of label-free liquid chromatography mass spectrometry to analyze the expression of multiple endogenous peptides and the potential to detect new putative peptide candidates in a developmental model.

## Background

Bird models have been instrumental to the overall understanding of neural sex differences and endocrine influences on brain development [1,2]. Japanese quail is a commonly used bird model for studying sex-specific brain development and behavior, especially the influence of the hormonal milieu during early brain development on sexual behaviour [3-5]. Sex-specific neural development has traditionally been associated with sex hormones produced by the gonads. Exogenous estrogen exposure before embryonic day 12 (ed12) causes demasculinization of the male sexual behaviour in Japanese quail [3,5], which shows the important role of sex hormones in brain differentiation. Several studies in songbirds have shown that there are exceptions to this classical model [6-9], implicating an intrinsic genetic influence on sex-specific neural development. Our previous studies on gene expression in early chicken [10] and quail embryos (ongoing) also indicate a sex-specific brain development that is at least partly independent of the influence of gonadal hormones. Quail ed12 corresponds roughly to chicken ed14 which is close to the developmental stage when gonad derived plasma testosterone levels peak (~ed13.5) in male embryos and the endocrine hypothalamic-pituitary-gonadal (HPG) axis becomes established [11-13]. Endogenous gonad derived plasma estradiol levels increase gradually in female chicken embryos until ~ed13.5 where they increase markedly [14,15]. Male chicken embryo plasma estradiol levels are significantly lower than female levels at all stages although the differences become more pronounced after ed13.5 [15]. The establishment of the male HPG axis is believed to be dependent on both hypothalamic gonadotropin-releasing hormone release and opioid peptide regulation [12]. Several endogenous peptides, including opioid peptides, are messengers with sex-specific activity, some of them being stored and secreted in an endocrine-like manner [16]. Opioid peptide dependent pain and analgesia [17] are well known examples of neural sex differences. Pain and analgesia are conditions that are sensitive to neonatal alterations in gonadal steroid hormones [18]. Studies using the opioid receptor antagonist naloxone on the medial preoptic area (POM) in the anterior diencephalon point to a role for opioid peptides in the regulation of both avian and mammalian sexual behavior [19-21].

A group of neuropeptides may have the same pre-cleavage protein precursor in common, meaning that one gene may code for several peptides with different functions. Although genome information from different species has facilitated prediction of putative precursor cleavage sites and peptide/precursor amino acid sequences, little is known about the expression levels of different peptides under different conditions. This is especially true regarding the peptidome content in the developing brain. The sequencing of the chicken genome has enabled prediction and expression analysis of neuropeptides in avian models such as Japanese quail. Neuropeptidomics is the technological approach for detailed analysis of endogenous peptides in the brain. Recently, a novel approach to study a large number of neuropeptides using nL/min flow liquid chromatography (nanoLC) electrospray ionization mass spectrometry was developed to investigate the endogenous neuropeptide content of brain tissue samples from rats and mice [22-24].

The aims of this study were to characterize the endogenous peptide content of the diencephalon including the sexually dimorphic area POM in the developing quail brain and to analyze how its expression is related to age, sex and the effect of ethinylestradiol (EE_2_) exposure. Quail ed12 diencephalon was chosen as both the stage of development and the tissue are of interest in this context. Ed12 is at the end of the sensitivity period for embryonic estrogen exposure leading to behavioral alterations in male copulatory behavior [5] and likely in the time period when the male endocrine HPG axis becomes established and a more marked increase of female estradiol synthesis occurs. Quail ed17 is somewhat before hatching and a time where the male embryos lost their sensitivity to exogenous estradiol and their sex specific brain and neuroendocrine development has become established. The detection of neuropeptides and the quantification of their expression levels were done using liquid chromatography and ion trap mass spectrometry together with a label-free approach.

## Results

### Peptide identification

Japanese quail embryonic diencephalon protein samples from 38 individuals were separated by molecular weight, obtaining < 10 kD samples. These samples were then examined using nano LC coupled to an ESI-LTQ MS system or an LTQ-FTICR system. A total of 65 peptides were identified (Table 1) of which 38 (Table 2) were present in a sufficient number of individuals to enable expression profiling (see below). Among the 65 peptides, 61 were derived from neuropeptides or neuropeptide precursor proteins. The four remaining peptides belonged to CRMP-2, FKBP5 (also called PPIA), Stathmin and Thymosin beta.

**Table 1:** Peptides identified by LTQ and FTICR-MSMS in quail diencephalon.

Precursor	Peptide	MH+	Sequence	Comment	PCA & PLS/DA^1^
Acyl-CoA binding protein	ODN peptide	1637.56	TVGDVNTDRPGMLDF		Yes
Cerebellin-1 pre.	Cerebellin-1	1632.85	SGSAKVAFSAIRSTNH		Yes
Cerebellin-2 pre.	Cerebellin-2	1620.80	SGSAKVAFSATRSTNH		
Cerebellin-4 pre.	Cerebellin-4	1451.70	ANSKVAFSAVRSTN		Yes
	Cerebellin-4	1267.36	SKVAFSAVRSTN		Yes
Gastrin-releasing peptide	GRP	1766.69	APLQPGGSPALTKIYPR		Yes
	GRP	1608.89	APLQPGGSPALTKIYP		
Gonadotropin-inhibiting hormone	GnIH propep	1743.83	SVPISLSQGVQESEPGM		
	GnIH-RP2	1723.97	SPLARSSIQSLLNLPQ		
	GnIH-RP2	1426.80	ARSSIQSLLNLPQ		Yes
Glucagon family neuropeptides	PACAP27	1573.73	HIDGIFTDSYSRY		
Neuropeptide Y (NPY) pre.	NPY	1443.81	SSPETLISDLLLR		
Prepronociceptin (PNOC) pre	Precursor	1226.82	AVASPLQVSELL		Yes
	Nociceptin	1981.07	YGGFIGVRKSARKWNNQ		Yes
	Nociceptin	1154.63	YGGFIGVRKSA		Yes
	Nociceptin	996.56	YGGFIGVRK		
	Neuropeptide1	1981.05	GSWPAARGVQ		Yes
	Neuropeptide2	1702.86	FSEFLKQYLGMSPR		Yes
	Neuropeptide2	1555.79	SEFLKQYLGMSPR		Yes
Proenkephalin A (PENK) pre	Precursor	2218.94	MDELYHPESEDEANGGEILA*		
	Precursor	2147.91	MDELYHPESEDEANGGEIL*		
	Precursor	2087.90	DELYHPESEDEANGGEILA*		
	Precursor	1972.44	ELYHPESEDEANGGEILA*		Yes
	Precursor	1901.84	ELYHPESEDEANGGEIL*		
	Precursor	1843.83	LYHPESEDEANGGEILA*		
	Precursor	1730.75	YHPESEDEANGGEILA*		
	Precursor	1659.72	YHPESEDEANGGEIL*		
	Precursor	1496.65	HPESEDEANGGEIL*		
	Precursor	1448.69	VGRPEWWLDYQ**		
	Precursor	1387.62	SPELEDEAKELQ*		Yes
	Precursor	1259.90	SPELEDEAKEL*		Yes
	Precursor	1203.57	ELEDEAKELQ*		
	Precursor	1074.99	LEDEAKELQ*		Yes
	MERF	877.40	YGGFMRF		Yes
	MERSL	930.45	YGGFMRSL		Yes
Proenkephalin B (PENK) pre	Precursor	1028.80	PKLKWDNQ		Yes
Protachykinin 1 (PPT) pre	C-term flanking peptide (CTFP)	1741.07	SLNSGSSERSIAQNYE		Yes
	C-term flanking peptide (CTFP)	1821.77	SLNSGSSERSIAQNYE	S6/7(p)	Yes
	C-term flanking peptide (CTFP)	1900.26	SLNSGSSERSIAQNYE	S6/7, S10(p)	Yes
	Substance P				Yes
	Neuropeptide K	947.42	DAGYGQISH		Yes
	Neuropeptide K	832.40	AGYGQISH		
	Neurokinin A	1133.87	HKTDSFVGLM	M-amide	Yes
Secretogranin-1 pre	Secretogranin1- propep	1725.81	QYDKMDQLAQLLNY	Pyroglutamic Acid	
	Secretogranin1	1357.54	IHEGEEGEAEEE		Yes
Secretogranin-2 pre	Secretoneurin	2064.99	TNEIVEEQYTPQSLATLE		
	Secretoneurin	1650.79	TNEIVEEQYTPQSL		Yes
	Secretoneurin	1537.70	TNEIVEEQYTPQS		
	Secretogranin2	1255.59	SGKLSFLEDEM		
	Secretogranin2	1124.55	SGKLSFLEDE		Yes
	Secretogranin2	880.478	SGKLSFLE		
Secretogranin-5 pre	C-terminal peptide (CTP)	1772.96	SVNPYLQGKRLDNVVA		Yes
	C-terminal peptide (CTP)	1701.63	SVNPYLQGKRLDNVV		Yes
Somatostatin pre	SMS-propep	1256.71	SLAAAAGKQELAK		
	SMS-14	1409.18	KNFFWKTFTSC		Yes
	SMS-14	1019.90	FWKTFTSC		Yes
	SMS-28	1226.61	SANSNPALAPRE		Yes
	SMS-28	1097.57	SANSNPALAPR		
	SMS-28	1139.58	ANSNPALAPRE		
Vasoactive intestinal peptide	VIP	1219.7	AVFTDNYSRF		Yes
CRMP-2		1210.81	APPGGRANITSLG		Yes
FKBP5		1712.77	ANAGPNTNGSQFFICTA		Yes
Stathmin		1358.74	ASGQAFELILGPR		
Thymosin beta		1566.84	SDKPDMAEIEKFDK		Yes

^1 ^Partial Least Square Discriminant Analysis * Propeptide to Met-enkephalin.

**Propeptide to Leu-enkephalin

**Table 2:** Differentially expressed peptides

Precursor	Peptide	Sequence	F-test FDR adj. p-value^1^	Age effect log2 fold change	Sex effect log2 fold change	EE2 effect log2 fold change	Interactions^2^
ACBP	ODN-peptide	TVGDVNTDRPGMLDF	p < 0.001	3.80***			p < 0.01
CBLN1	Cerebellin-1	SGSAKVAFSAIRSTNH	n.s.				n.s.
CBLN4	Cerebellin-4	ANSKVAFSAVRSTN	n.s.				n.s.
CBLN4	Cerebellin-4	SKVAFSAVRSTN	n.s.				n.s.
GRP	GRP	APLQPGGSPALTKIYPR	p < 0.001	1.08***	-0.59***		n.s.
GnIH	GnIH-RP2	ARSSIQSLLNLSQ	p < 0.001	3.41***	-0.94*		p < 0.01
PNOC	PNOC	AVASPLQVSELL	p < 0.001	1.62***			n.s.
PNOC	Nociceptin	YGGFIGVRKSARKWNNQ	p < 0.001	1.56***			p < 0.05
PNOC	Nociceptin	YGGFIGVRKSA	p < 0.01	0.75***		0.45*	p < 0.05
PNOC	Neuropeptide1	GSWPAARGVQ	p < 0.001	1.24***		0.29*	n.s.
PNOC	Neuropeptide2	FSEFLKQYLGMSPR	p < 0.001	1.17***			n.s.
PNOC	Neuropeptide2	SEFLKQYLGMSPR	p < 0.001	2.24***			n.s.
PENK	Precursor	ELYHPESEDEANGGEILA	n.s.				n.s.
PENK	Precursor	SPELEDEAKELQ	p < 0.001	0.92***			n.s.
PENK	Precursor	SPELEDEAKEL	p < 0.001	2.36***	0.57*		n.s.
PENK	Precursor	LEDEAKELQ	p < 0.001	1.76***			n.s.
PENK	Precursor	VGRPEWWLDYQ	p < 0.001	0.96***		0.51*	p < 0.05
PENK	MERF	YGGFMRF	p < 0.001	1.59***			n.s.
PENK	MERSL	YGGFMRSL	p < 0.001	0.95***		0.35*	n.s.
PENKB	Precursor	PKLKWDNQ	p < 0.01	2.36***			n.s.
PPT	CTFP	SLNSGSSERSIAQNYE	n.s.	0.66**			n.s.
PPT	CTFP (1P)	SLNSGSSERSIAQNYE	p < 0.001	1.28***			n.s.
PPT	CTFP (2P)	SLNSGSSERSIAQNYE	p < 0.05	0.92***			n.s.
PPT	SP	RPRPQQFFGLM	p < 0.001	1.80***			n.s.
PPT	NPK	DAGYGQISH	n.s.			-0.99***	p < 0.05
PPT	NKA	HKTDSFVGLM	p < 0.001	1.78***			n.s.
SCG1	SCG1	IHEGEEGEAEEE	n.s.				n.s.
SCG2	Secretoneurin	TNEIVEEQYTPQSL	n.s.	0.40*			n.s.
SCG2	SCG2	SGKLSFLEDE	p < 0.001	2.40***			p < 0.05
SCG5	CTP	SVNPYLQGKRLDNVVA	p < 0.001	1.12***		0.18*	n.s.
SCG5	CTP	SVNPYLQGKRLDNVV	p < 0.001	2.64***			n.s.
SMS	SMS-14	KNFFWKTFTSC	p < 0.001	2.62***			p < 0.05
SMS	SMS-14	FWKTFTSC	p < 0.001	2.70***			n.s.
SMS	SMS-28	SANSNPALAPRE	p < 0.001	1.03***			n.s.
VIP	VIP	AVFTDNYSRF	p < 0.001	2.76***			n.s.
CRMP-2		APPGGRANITSLG	p < 0.01			-0.82**	p < 0.01
PPIA/FKBP12		ANAGPNTNGSQFFICTA	p < 0.001	-0.55***	-0.29*		n.s.
Thymosin beta		SDKPDMAEIEKFDK	n.s.				n.s.

^1^. The F-test describes the significance of the linear model that incorporates the three parameters in this study: age (ed12 and ed17, sex (male and female), and exposure (controls and EE2 exposed). There are three levels of statistical significance for the parameter effects: p < 0.05 (*), p < 0.01 (**) and p < 0.001 (***). A minus sign (-) in the age effect column means that the peptide is more highly expressed in ed12 than ed17. A minus sign (-) in the sex effect column means that the peptide is more highly expressed in males compared to females. A minus sign (-) in the EE2 effect column means that the peptide is more highly expressed in controls compared to exposed. ^2^. The significance of the interaction effects was calculated by Walds F-test. n.s. means non-significant.

### Expression profiling

**Figure 1:**
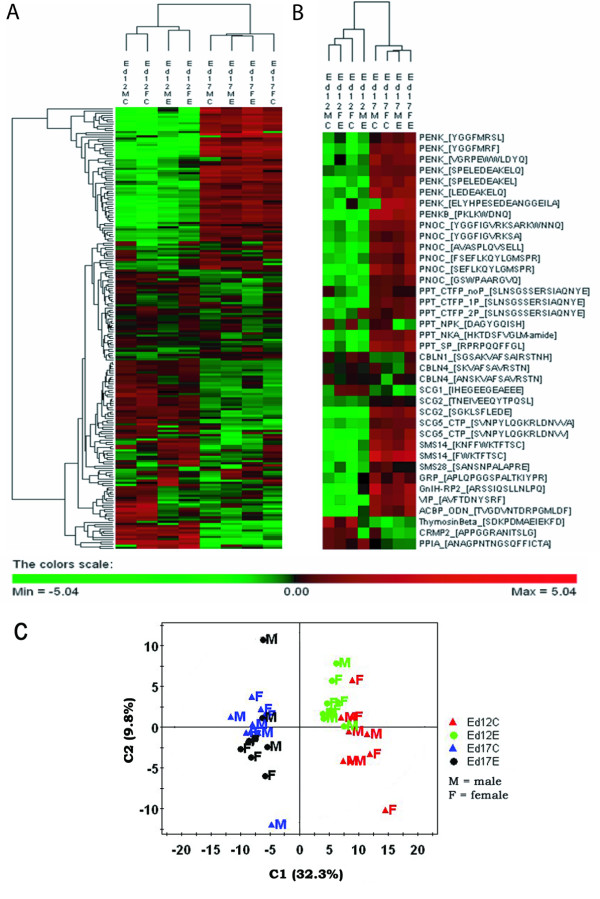
**Unsupervised hierarchical clustering and principal component analysis**. (**A**) Visualization of group differences (ed12MC, ed12FC, ed12ME, ed12FE, ed17MC, ed17FC, ed17ME, ed17FE) for 204 peptides, using two-way clustering of the log2 ratio (*Xclass-Xtot*) between the group median value (X_class_) and the total median value for every peptide (X_tot_). (**B**) One-way clustering of 38 identified peptides. Positive values are colored red, negative are green. (**C**) Principal component analysis using two components (C1, C2). The last letter(s) in the group definitions ed12/17XX stand for M = male, F = female, C = controls, E = ethinylestradiol exposed. # designates the peptide numbers in the data set.

A subset of the LTQ-MS samples were divided into eight groups by sex (male vs female), age (ed12 vs ed17) and EE_2_-exposure (tissues collected from ed12 and ed17 males and females) with four to five samples in every group. The samples were ed12 male controls (n = 5), ed12 female controls (n = 4), ed12 male EE_2 _exposed (n = 4), ed12 female EE_2 _exposed (n = 4), ed17 male controls (n = 4), ed17 female controls (n = 4), ed17 male EE_2 _exposed (n = 4) and ed17 female EE_2 _exposed (n = 5). The alignment of peptide spectra was performed using the DeCyder MS software. Expression data for 204 peptides, whereof 38 identified, was first analyzed by unsupervised methods (hierarchical clustering, principal component analysis, PCA) and then by a supervised method (Partial Least Squares Discriminant Analysis, PLS/DA). A clear separation between ed12 and ed17 peptide expression was visible in a hierarchical clustering (Figure 1A) of all eight experimental groups with the majority of the identified peptides being more highly expressed in ed17 than in ed12 diencephalon (Figure 1B). A similar pattern was detected with PCA (Figure 1C, additional file 1).

**Figure 2:**
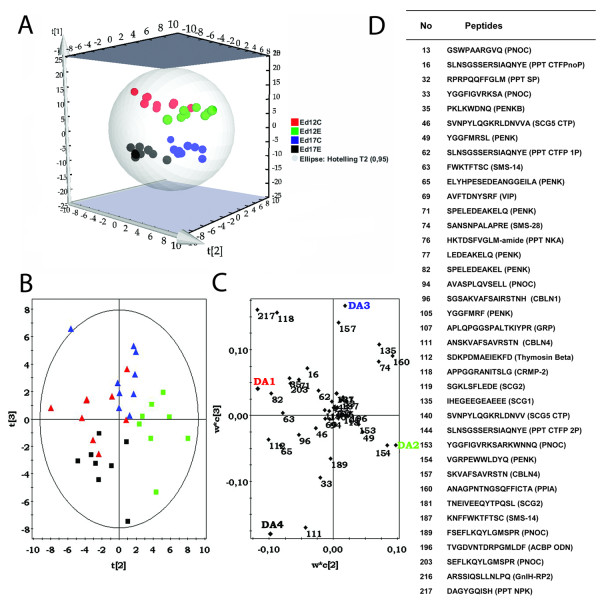
**PLS/DA on age and EE_2 _exposure using 204 quail peptides**. (**A**) Three-dimensional score plot for all observations using the first three components (explaining ~75% of the data) in a model on age and EE_2 _exposure effects. The PLS/DA model uses the dummy variables DA1 (ed12C, red color), DA2 (ed12E, green color), DA3 (ed17C, blue color), and DA4 (ed17E, black color). In (**B**), the second and third components are combined in a score scatter plot, separating controls (red and blue triangles) from EE_2 _individuals (green and black boxes). The influence of identified peptides on the positioning of samples in the score scatter plot is seen in the loading scatter plot (**C**). The 38 identified peptides in the loading plot are listed in (**D**). Score/loading axes for DC2 and DC3 are designated t(2)/w*c(2) and t(3)/w*c(3) respectively. ed12/17C designates ed12/17 controls and ed12/17E designates ethinylestradiol exposed.

**Figure 3:**
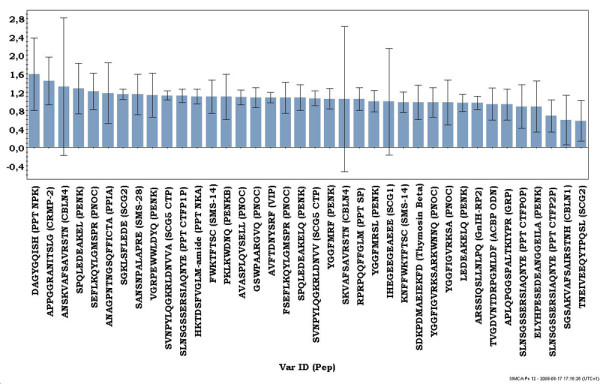
**Virtual importance in the projection plot (VIP)**. The VIP shows the role of identified peptides in the separation of the four age and EE_2 _exposure PLS/DA classes (ed12C, ed12E, ed17C, ed17E). The preprotachykinin-1 neuropeptide K (NPK) is the top candidate among identified peptides for being affected by both age and EE_2 _exposure. Values above one are the most relevant for the separation of all classes. Unidentified peptides are not shown. Error bars designate confidence intervals derived by jack knifing.

Both analyses indicate a weak separating effect of EE_2 _exposure in ed12 samples. PLS/DAs were conducted to explore the maximized separation between groups regarding age, sex and EE_2 _exposure. There were no successful models separating all three parameters (sex, age and EE_2_) leading to a PLS/DA model for age and EE_2 _exposure (Figure 2, additional file 2). The effect of EE_2 _exposure was more distinct on ed12 than on ed17 (Additional file 3). A three-dimensional PLS/DA score plot (Figure 2A) shows the separation of age (ed12 and ed17) and EE_2 _exposure (controls, C and exposed, E), independently of sex (ed12C, ed12E, ed17C, ed17E), with the second and third component being most effective in separating controls from EE_2 _exposure (Figure 2B-D). Next, a virtual importance in the projection (VIP) (Figure 3) was plotted to see how much the identified and unidentified peptides influence the different class separations in the PLS/DA. The preprotachykinin 1 peptide neuropeptide K (PPT NPK, VIP 1.59) was most important for overall separation among the identified peptides, followed by a peptide derived from CRMP-2 (VIP 1.45). These two peptides also display a significantly interaction effect when using moderated F-statistics (see below) (Table 2). Four unidentified peptides (VIP 1.6-1.85) were more relevant than NPK (data not shown). NPK, the top PLS/DA candidate for overall effects on classes based on age and EE_2 _exposure, was also the most influential among ed12 samples (see Additional file 3).

**Figure 4:**
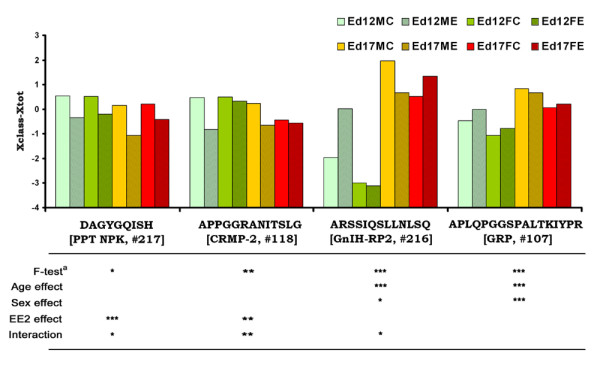
**Candidate diencephalon peptides sensitive to sex differences or EE_2_**. Visualization of the differences of candidate peptides (NPK, CRMP-2, GnIH-RP2, and GRP) between all eight groups (ed12MC, ed12FC, ed12ME, ed12FE, ed17MC, ed17FC, ed17ME, ed17FE) as calculated (*Xclass-Xtot*) for the hierarchical clustering in figure 1. The stars designate significance levels (*** p < 0.001, ** p < 0.01 and * p < 0.05) for the different factors in the model. ^a ^FDR adjusted p-value significance for F-test. Interaction effects were tested using Wald F-test. The last letters in the group definitions ed12/17XX stand for M = male, F = female, C = controls, E = ethinylestradiol exposed.

Multivariate analysis methods such as PCA and PLS/DA are efficient for illustrating general trends and patterns in complex data but more limited in determining the significance/effect for single factors (peptides). An F-test was used to detect statistically significant (adjusted p < 0.05) differences in singular peptide expression between groups regarding the response variables age, EE_2 _exposure and sex. The age differences among identified peptides were dominated by a general upregulation between ed12 and ed17 (Figure 1B, Table 2). There were a few exceptions, such as NPK and the cerebellin derived peptides (Table 2). Only few peptides were significantly affected by either sex or EE_2 _exposure (Table 2). The top EE_2 _candidates were NPK and the CRMP-2 derived peptide (Figure 4). This was also indicated by their localization (peptides #118, 217) in the PLS/DA VIP (Figure 3) and the loading scatter plot (Figure 2C-D) where they are most prominent in separating control samples (ed12C, ed17C) from EE_2 _samples (ed12E, ed17E). The CRMP-2 peptide displays EE_2 _induced downregulation of expression in males at ed12 and ed17 (in average ~57%), whereas NPK is downregulated in both males and females at ed12 and ed17 (in average 50%). Peptides derived from gastrin-releasing peptide (GRP; APLQPGGSPALTKIYPR) and gonadotropin inhibitory hormone related peptide 2 (GnIH-RP2) were the top candidates for sex specific differences (Figure 4). GRP was more highly expressed (in average 66%) in all male groups compared to their respective female groups independent of EE_2 _exposure (Figure 4) and GnIH-RP2 was more highly expressed (in average 52%) in ed12 males and in ed17 control males.

### Secretogranins

**Figure 5:**
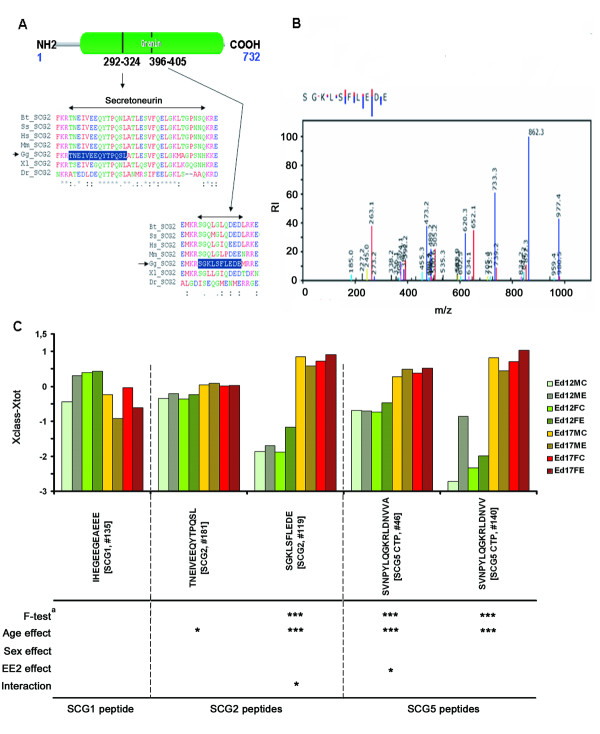
**Japanese quail secretogranins**. (**A**) Overview of avian secretogranin-2 (based on chicken sequence XP_422624) showing its granin protein domain and the position and multiple sequence alignment to other species for two identified peptides: TNEIVEEQYTPQSL (Secretoneurin peptide) and SGKLSFLEDE. Bt is *Bos taurus*, Ss is *Sus scrofa*, Hs is *Homo sapiens*, Mm is *Mus musculus*, Gg is *Gallus gallus*, Xl is *Xenopus leavis*, and Dr is *Danio rerio*. (**B**) MS/MS fragmentation graph for SGKLSFLEDE in GPM browser. RI stands for relative intensity. **C**, Visualization of expression differences between the groups as calculated (*Xclass-Xtot*) for the hierarchical clustering in figure 1. The stars designate significance levels (*** p < 0.001, ** p < 0.01 and * p < 0.05) for the different factors in the model. ^a ^FDR adjusted p-value significance for F-test. Interaction effects were tested using Wald F-test. The last letters in the group definitions ed12/17XX stand for M = male, F = female, C = controls, E = ethinylestradiol exposed.

Peptides from three secretogranins (SCG1, SCG2, and SCG5) were identified (Table 1). Two out of six SCG2 peptides were detected in all samples. The two SCG2 derived peptides (TNEIVEEQYTPQSL and SGKLSFLEDE) behave in different manners as seen by F-test statistics (the former being non-significant and the later significant, F-test statistics, adjusted p < 0.05). Both show an age effect, becoming more highly expressed in ed17 but only SGKLSFLEDE shows a significant interaction effect and approaches significance for a treatment effect (t-test p-value 0.062). SGKLSFLEDE is located between two basic amino acid cleavage sites (KR, RR) and corresponds to human SCG2 285-295 (Figure 5). The full peptide probably includes an additional C-terminal methionine (SGKLSFLEDE *M*) if one considers the full sequence between the cleavage sites. Such a peptide was also discovered (Table 1) but its presence across all samples was insufficient for matching purposes. The amino acid sequence of the secretogranin-1 (chromagranin B) peptide (IHEGEEGEAEEE) is less conserved between birds and mammals and the peptide as such seemingly unaffected by age. The peptide is located in a non-conserved sequence region of secretogranin-1 (data not shown) and no deduction about relevance or functionality was possible.

### Protachykinins

**Figure 6:**
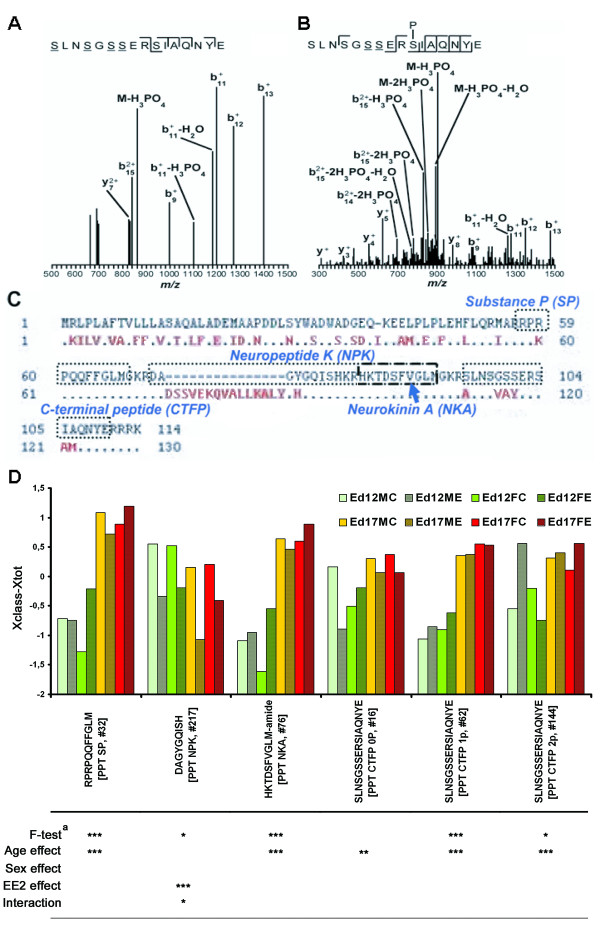
**The preprotachykinin-1 peptides**. SLNSGSSERSIAQNYE is similar to the C-terminal flanking peptide (CTFP) in mammalian protachykinin 1. Two previously unknown serine phosphorylations at positions S6 or S7 and S10 were discovered. (**A**) FTICR derived MS/MS spectra for single phosphorylated form (Mw 1820) at S6 or S7 and (**B**) S10 (Mw 1900.3). Amino acid sequence alignment (**C**) between chicken (continuous sequence in black color) and human (red text) preprotachykinin 1 precursor shows the differences between birds and mammals in the detected tachykinins, especially neuropeptide K (NPK). (**D**) Visualization of differences between the groups as calculated (*Xclass-Xtot*) for the hierarchical clustering in figure 1. The stars designate significance levels (*** p < 0.001, ** p < 0.01 and * p < 0.05) for the different factors in the model. ^a ^FDR adjusted p-value significance for F-test. Interaction effects were tested using Wald F-test. The last letters in the group definitions ed12/17XX stand for M = male, F = female, C = controls, E = ethinylestradiol exposed.

Among the peptides identified, several were part of the protachykinin 1 precursor (designated PPT). Representative peptide sequences for all peptide forms (substance P [SP], NPK, neurokinin A [NKA] and C-terminal flanking peptide [CTFP]) except neuropeptide gamma were detected. FTICR-MS enabled us to detect two previously unknown phosphorylations on the C-terminal flanking peptide (CTFP), on S6 or S7 (single phosphorylated; Figure 6A) and S10 (double phosphorylated with S6/7; Figure 6B). The detection of the truncated NPK sequence DAGYGQISH (Figure 6C) shows that quail has the same sequence deletion in its Protachykinin gene as seen in chicken compared to mammals. The log2 ion intensity levels for non-phosphorylated and single-phosphorylated CTFP isoforms were relatively similar (log2 ion intensity ~18-19) across all groups whereas the expression of the double-phosphorylated isoform was much lower (log2 ion intensity ~12-13).

## Discussion

There is very little known about neuropeptide expression patterns and regulation in the developing embryonic brain. This is the first peptidomics study on neuropeptide expression in the developing avian brain. A total of 65 peptides from the embryonic quail diencephalon were identified (Table 1). Using the minimum matching criteria of max one missing sample per group resulted in 204 matched peptides, including 38 identified. These were plotted for differences related to age (ed12 and ed17), sex and EE_2 _exposure. The general patterns in the data set were explored using unsupervised and supervised multivariate analysis and unsupervised hierarchical clustering. These methods demonstrated a clear age separation between ed12 and ed17 individuals. The fact that most identified peptides were more highly expressed in ed17 compared to ed12 was not representative for all plotted peptides (Figure 1A-B, Table 2). The PCA and hierarchical clustering also revealed a less distinctive but still discernable separation of control and EE_2 _exposed individuals, although mainly at ed12. Overall, the data indicates that the ed12 quail diencephalon shows a more distinct EE_2 _sensitive and sex peptide expression pattern as compared to ed17 (Figure 1, Additional file 3). If true, it may be correlated with the establishment of the HPG axis occurring at this developmental stage in male quail embryos [12,13]. No overall sex-specific patterns were detected, which was somewhat unexpected considering the sexual dimorphic nature of the diencephalon and the large sex differences observed in birds on the mRNA level [10,25,26]. The mRNA differences are mainly based on gene dosage effects from the Z chromosomes (males ZZ; females ZW). Only one peptide (GRP) in our data was derived from a gene located on the Z chromosome, possibly explaining the absence of large scale sex differences. Interestingly, the sex chromosome Z derived GRP peptide was the top candidate for sex specific peptide expression and was significantly more highly expressed in males compared to females in both controls and EE_2 _exposed individuals. The absence of general sex differences was also unexpected in relation to the detection of candidates for the demasculinizating EE_2 _effects on the copulatory behavior in adult males following embryonic estrogen exposure [5,27].

Most of the identified peptides were part of larger neuropeptide precursor proteins. Peptides derived from the same secretogranin-2 precursor displayed expression differences indicating peptide specific regulation. Both peptides were more highly expressed in ed17 but only SGKLSFLEDE was significantly affected over all groups (F-test statistics, adjusted p < 0.001) with a significant interaction effect and near significant EE_2 _treatment effect (p-value 0.062). The positioning of SGKLSFLEDE between basic amino acid pairs (KR and RK, Figure 5A) and its expression pattern compared to Secretoneurin indicates that it possesses a specific biological function and makes it a potential new avian neuropeptide candidate. Besides SGKLSFLEDE, two other forms (SGKLSFLE and SGKLSFLEDEM) were also detected, although not in all the samples. The later, SGKLSFLEDEM is probably the complete peptide as it represents the complete sequence between the two cleavage sites in the precursor protein.

Previous studies have demonstrated that opioid peptides are involved in aggressive and sexual behaviors in adult Japanese quail [21,28] and also in the establishment of the male chicken embryonic HPG axis [12]. We detected a large number of proenkephalin precursor (PENK) derived peptides (Table 1, Additional file 4), although the majority were propeptides located in front of the opioid peptides. None of the identified opioid peptides (MERF or MERSL) differed significantly between groups except with regard to age. The detection of Met-Enkephalin (Met-Enk) and Leu-Enkephalin (Leu-Enk) propeptides indicates that Met-Enk and Leu-Enk also are expressed in the developing diencephalon. Their absence may be explained by the fact that their short sequences make it more difficult to detect them with LC-MS. The presence of the complete Leu-Enk propeptide (VGRPEWWLDYQ) in all groups and it being significantly affected by age and EE_2 _exposure plus exhibiting interaction effects (Table 2) may indicate that Leu-Enk also is differentially expressed. Among the peptides derived from another large neuropeptide precursor protein, Preprotachykinin 1, we identified two previously unknown phosphorylations on the C-terminal flanking peptide (CTFP), on S6 or S7 (single phosphorylated; Figure 6A) and S10 (double phosphorylated with S6/7; Figure 6B). Quail SP possesses the same Arg for Lys substitution at the third position compared with humans as previously described in chicken [29], alligator [30] and Burmese python [31] whereas quail NKA is identical to chicken, alligator, Burmese python and human NKA [31]. NKA is the ten amino acid long C-terminal part of NPK (36 amino acids) in mammals. The LC-MS detection and identification of the truncated NPK sequence DAGYGQISH (Figure 6C) shows that quail has the same sequence deletion in its Protachykinin gene compared to mammals as seen in chicken. Antisera against a mammalian NPK fragment which encompasses some of the chicken NPK sequence shows immunoreactivity in the developing chicken embryonic spinal cord which weakens after hatching [32]. The nine amino acid long quail NPK could work in a functionally inert propeptide fashion to NKA in quail considering that NPK is converted into NKA in mammals [33]. It is not clear if the truncated avian NPK has a biological function although it is indicative that it is the top candidate for influencing overall separation in the multivariate datasets with regard to age and EE_2 _exposure, especially at ed12 (Figures 2 and 3, Additional file 3) and becomes significantly downregulated in all groups after EE_2 _exposure (Figure 4, Table 2). The different age dependent expression patterns seen in our data for NPK and NKA (NPK is unaffected by age whereas NKA is significantly upregulated) can also be interpreted as involvement in separate biological processes during diencephalon development. NPK is known to regulate gonadotropin secretion in the mammalian hypothalamus [34], and our data indicates that this may also be true for avian diencephalon development.

There were only few peptides exhibiting sex differences in expression, the top candidates being the GRP and GnIH-RP2 peptides. The first 17 amino acids of GRP was as previously mentioned the only ChrZ-derived peptide detected in our data and it was generally more highly expressed in males compared to females (in average 66%). GRP is an interesting candidate for sex specific brain development as expression of its receptor has been related to the layering of cells in the chick telencephalon, optical tectum and cerebellum [35]. The GnIH-RP2 peptide is part of the gonadotropin inhibitory hormone (GnIH) precursor protein (UniProt GNIH_COTJA) whose main peptide (GnIH) inhibits gonadotropin release in quail and is strongly expressed in all avian species studied so far [36,37]. The GnIH-RP2 peptide was significantly differentially expressed between groups with a clear age effect from ed12 to ed17 (Figure 4, Table 2). It also displayed significant sex and interaction effects. It should be noted that the identified sequence (ARSSIQSLLNLSQ) is actually somewhat longer than the calculated GnIH-RP2 sequence (SSIQSLLNLSQRF) and does not posses the characteristic C-terminal RF-amide. The observed peptide may be part of a larger propeptide-GNIH-RP2 peptide sequence that has become degraded at both the N and C-terminal. The GnIH precursor protein contains a neuropeptide cleavage site (RxnR where n = 2, 4 or 6) [38] adjacent to a somewhat longer sequence (*SPL*ARSSIQSLLNLSQ) of the peptide in question (Table 2, Additional file 4). Unlike GnIH itself, there is little known about the role of GnIH-RP peptides. Although this may be the case, the overall expression pattern of this particular peptide implies a biological role in avian embryonic diencephalon development for GnIH-RP2. Both GnIH-RP peptides posses affinity for the GnIH receptor [39], indicating that GnIH-RP2 is involved in the regulation of gonadotropin release from the developing pituitary. NPK and GnIH-RP2 are both potential regulators of gonadotropin regulation during diencephalon development and the male HPG axis formation. The EE_2 _effects on GnIH-RP2 expression are not very influential/statistical significant in either type of data analysis (multivariate or univariate) and therefore not conclusive. Still, the fact that EE_2 _also seems to modulate GnIH-RP2 expression (Figure 4) is intriguing considering that the NPK expression is EE_2 _sensitive. It could indicate that the male quail embryonic window of sensitivity (regarding adult male copulatory behavior) before and around ed12 to exogenous EE_2 _exposure is partly influenced by an altered gonadotropin regulation and HPG axis formation.

It is important to note that this study is foremost of hypothesis generating nature. We have analyzed the expression of a large number of neuropeptides (including detailed amino acid sequence information) under different circumstances in the developing avian brain in a manner that is impractical or impossible for the more traditional immunoassay methods. Omics data is valuable when the experimental design includes several variables (in our case age, sex and EE_2 _exposure corresponding to three variables or eight experimental groups) but requires at the same time an increasing number of samples and makes the analysis and presentation of the results more difficult. Further studies are needed to better analyze the role of the individual neuropeptides during development using for instance more data points for age or the addition of aromatase inhibitors to better deduce the impact of EE_2_. The absence of neuropeptides such as vasotocin was for instance unexpected although this does not provide any conclusions on their presence or role in embryonic brain development. The absence of proof is not the proof of absence. Peptides such as vasotocin contain two cysteine amino acids and can contain a disulfide bridge, a property that makes them more difficult to fragment and identify with MS/MS. Additional peptides could for instance be detectable in less hydrophilic sample preparation buffer settings than the weakly acidic 0.25% acetic acid buffer used in this study. Future peptidomics studies such as this study may provide even more data when other sample preparations strategies or additional mass spectrometry systems besides LTQ-MS are added.

## Conclusion

To summarize, we report the first large scale neuropeptidomic analysis of the developing avian diencephalon at ed12 and ed17 in Japanese quail, a common model for studying sex-specific neural development. The peptidome was analyzed in relation to age, sex and EE_2 _treatment at ed3. Both age and EE_2 _treatment affected the quail peptidome, without any clear discernible influence of gender. A potential new avian neuropeptide candidate was discovered in the secretogranin-2 precursor protein and previously unknown phosphorylation sites in the protachykinin C-terminal flanking peptide identified. GnIH-RP2 and GRP are likely to be differentially expressed in a sex specific manner and NPK in an EE_2 _sensitive manner during embryonic brain development in Japanese quail. Our approach shows the strength of labeling-free LC-MS peptidomics and how to better characterize multiple known and unknown endogenous peptides in the developing organism.

## Methods

### Embryos and sample collection

Fertilized Japanese quail (*Coturnix coturnix japonica*) eggs were obtained from a local breeder. The eggs were incubated at 37.5°C and 60% relative humidity and turned every 3 h. On day three of incubation, eggs were injected with 300 ng ethinylestradiol (EE_2_) each. The EE_2 _was dissolved in an emulsion of peanut oil, lecithin, and water [40]. Controls received emulsion only. The holes in the shells were sealed with melted paraffin wax and the eggs were returned to the incubator. The diencephalon was excised as quickly as possible from embryonic brains on ed12 and ed17 and was placed in pre-weighed Eppendorf tubes. All samples were quickly frozen in liquid nitrogen, and stored at -80°C. These samples were then used for neuropeptidomic analysis. A tissue sample from each embryo was also collected for DNA isolation and genetic sexing according to a PCR-based method [41] in which intron sequences of different lengths in the W-linked gene CHD1W (females) and Z-linked gene CHD1Z (both sexes) are amplified. All animal experiments were approved by the local Ethics Committee for Animal Research, Uppsala, Sweden (reference number C 198/6).

### Sample preparation

Each frozen tissue sample was weighed and then placed on aluminum foil and put into the Denator Stabilizer instrument (prototype acquired from Denator Biotechnology, Gothenburg, Sweden) for heat denaturation for 45 seconds to protect the samples from post mortem protein degradation [42]. The order for denaturation of the samples was randomized. The tissue samples were then transferred to low-retention Eppendorf tubes and suspended in pre-chilled extraction solution (0.25% acetic acid; 0.2 mg tissue/μl) and homogenized by sonication (Vibra cell 750, Sonics & Materials Inc., Newtown, CT, USA) for 30 seconds [22,23]. Each sample suspension was then centrifuged at 20,000 g for 30 minutes at 4°C to remove insoluble material. The supernatant was transferred to Microcon 10 kDa cut-off spin columns (YM-10, Millipore, Bedford, MA, USA) and centrifuged for 45 minutes at 14,000 g at 4°C. The resulting peptide filtrate was then frozen at -80°C until analysis.

### Experimental design

All samples were run in a complete randomized block fashion with a total of five blocks (Table 3). Each block included replicates of the same sample (Q52) that was run repeatedly (six times, designated 52a-f). This resulted in a total of 42 runs, using 37 unique samples.

**Table 3:** Sample annotation and run order design

Run order	Sample Id	Age	Sex	C/T	Blocks
1	52a	Ed17	Female	C	B1
2	65	Ed17	Male	T	
3	30 *	Ed12	Female	C	
4	64	Ed17	Female	T	
5	10 *	Ed12	Male	T	
6	27	Ed12	Male	C	
7	4	Ed12	Female	T	
8	52b	Ed17	Female	C	
9	3	Ed12	Male	T	B2
10	23	Ed12	Male	C	
11	79	Ed17	Male	T	
12	9	Ed12	Female	T	
13	48	Ed17	Male	C	
14	78	Ed17	Female	T	
15	26	Ed12	Female	C	
16	54	Ed17	Female	C	
17	52c	Ed17	Female	C	
18	12	Ed12	Male	T	B3
19	49	Ed17	Male	C	
20	57	Ed17	Female	C	
21	24	Ed12	Male	C	
22	19	Ed12	Female	C	
23	72	Ed17	Male	T	
24	80	Ed17	Female	T	
25	2	Ed12	Female	T	
26	52d	Ed17	Female	C	
27	66	Ed17	Male	T	B4
28	6	Ed12	Male	T	
29	74	Ed17	Female	T	
30	29	Ed12	Male	C	
31	21	Ed12	Female	C	
32	55	Ed17	Female	C	
33	51	Ed17	Male	C	
34	7	Ed12	Female	T	
35	52e	Ed17	Female	C	
36	1	Ed12	Male	T	B5
37	68	Ed17	Female	T	
38	56	Ed17	Male	C	
39	62 ^*	Ed17	Female	C	
40	22	Ed12	Female	C	
41	25	Ed12	Male	C	
42	52f	Ed17	Female	C	

* Samples that subsequently were removed as outliers.

### MS analysis

Five μl of each sample was desalted on a Nano-Precolumn (LC Packings, Amsterdam, Netherlands) using Ettan MDLC (GE Healthcare). The peptides were then separated by a 40 minutes long run with a gradient from 3 to 80% acetonitrile in 0.25% acetic acid on a 15 cm long Proxeon nanoESI emitter (75 μm inner diameter, 360 μm outer diameter) packed in house with Reprosil-Pur C18 3 μm resin (Dr. Maisch GmbH, Ammerbuch-Entringen, Germany). At a flow rate of approximately 150 nl/min the peptides were electro sprayed into a linear ion trap mass spectrometer (LTQ, Thermo Electron, San Jose, CA, USA). The spray voltage was 1.8 kV, ES source capillary temperature was 160°C, and 35 units of collision energy were used to obtain peptide fragmentation tandem MS mode (MSMS). To increase the number of identifications, eight μl of different samples were also applied to an Agilent 1100 nanoflow system coupled to a 7-tesla hybrid LTQ Fourier transform ion cyclotron resonance (FTICR) MS (Thermo Electron, Bremen, Germany) equipped with a nanoelectrospray ion source (Proxeon Biosystems, Odense, Denmark) as described previously [43]. Mass spectrometric experiments were performed using unattended data-dependent acquisition in which the mass spectrometer automatically switches between a high resolution survey scan (r 100.000), (accuracy 5 ppm) followed by consecutive ECD and CAD fragmentation (r 25.000), (accuracy 0.02 Da) of the two most abundant multiply charged peptides eluting at this moment from the nano-LC column. Additional experiments were performed where the precursor masses were as previously detected with high mass accuracy (accuracy 0.02 Da), but the fragmentation was performed solely in the LTQ (accuracy 0.5 Da). Highmass accuracy spectra were deisotoped and filtered using the peptide window approach [43]. Detection of phosphorylated peptides was performed by PhosTShunter [44]. Raw MS data associated with the study may be downloaded from ProteomCommons.org Tranche network https://proteomecommons.org/index.jsp using the following hash:

Vui+Q2v4uXpRm2sWc5j8N3aNn+JISmG2RW9gnNMx0hBsse7DGTF3s7e+ci3lpmHUVsTuOKvvQTerSKqe5X3uzYSGbaUAAAAAAAAYWg==

### Peptide identification

The raw LTQ-MS data was converted to dta files by Xcalibur 1.4 SR1 and assembled by an in-house developed script to Mascot generic files. These files were searched against downloaded peptide rodent databases from the SwePep endogenous peptide database [45] and a precleaved chicken protein database derived from the NCBI RefSeq database. The pre-cleavage was performed as described by Fälth et al [24]. Estimation of false positives was conducted by searching all spectra against reversed databases [24]. Searches using MASCOT [46] and X! Tandem [47] (included in the Global Proteome Manager, GPM) against the previously mentioned databases were performed while checking for potential modifications (M oxidation, C-terminal amidation, Q/N deamidation, STY phosphorylations and N-terminal acetylation). Fragment mass error tolerance for both X! Tandem and MASCOT was set at 0.7 Da and the parent mass error was set to ± 1.5 Da. Two phosphorylated peptides were detected and characterized with FTICR MS and MASCOT as described above.

### LC-MS image analysis

All LTQ-data files were imported into the DeCyder software (version 2.0) for peak detection (PepDetect) and spot matching between samples (PepMatch). PepDetect parameters for peak detection were "Typical peak width: 0.2 min", "Ion-trap mass resolution: 0.7 u", "charge states: 1-10". All files were background corrected using a smooth surface model. Identities from the MASCOT and X! Tandem searches were incorporated into the DeCyder image files. Peaks that were not present after the initial DeCyder spot detection were added manually for all files.

### Removal of outliers

MS-data file outliers (see also table 3) were visually detected by the presence of increased protein degradation (Q62), i.e., more peptide peaks, or general low ion intensity (Q10 and Q30), the later by PCA analysis (see below). Removal of sample outliers left 39 MS-data files, of which six were technical replicates (ed12 female control; Q52a-Q52f) to each other, corresponding to 34 unique samples.

### Data normalization and analysis

Sample Q62 was removed from the data set before data normalization and analysis due to increased degradation. Ion intensity peptide match data was then exported for further data analysis. The resulting data was log2 transformed. In order to correct for global intensity differences between peptide runs the data was normalized in two steps as previously described [48]. A linear regression was fitted for each individual run to a median run that were constructed of all median peptide values for peptides that were matched in > 50% of all runs. Based on the linear regression equation new values were predicted for each run. A locally-weighted polynomial regression (Lowess) was then fitted for each matched peptide against the run order and the mean value across all runs were added to retain the native intensity dimension. For each matched peptide a proportion of 0.5 neighbors (runs), weighted by their distance to the measurement, were used for controlling the smoothness of the fit.

The data was grouped into eight groups: ed12 male controls (ed12MC, n = 5), ed12 female controls (ed12FC, n = 5), ed12 male EE_2 _(ed12ME, n = 5), ed12 female EE_2 _(ed12FE, n = 4), ed17 male controls (ed17MC, n = 4)), ed17 female controls (ed17FC, n = 4), ed17 male EE_2 _(ed17ME, n = 4), and ed17 female EE_2 _(ed17FE, n = 5) (Table 4). A minimum matching cut-off (max one missing file per group) resulted in 204 peptides. All 204 peptides had been manually inspected to control for mismatching. An unsupervised multivariate analysis by principal component analysis (PCA) using the SIMCA-P software (version 12, Umetrics AB, Umeå, Sweden) indicated previously mentioned Q10 (ed12ME) and Q30 (ed12FC) to be outliers which led them to be removed. Characterization of normalized log2 peptide ion intensities was performed by hierarchical clustering in the PermutMatrix program [49] using complete linkage and Euclidian distance as the distance metric. To better visualize age differences, the median value from the log2 ion intensities for each peptide and group (x_class_: ed12MC, ed12FC, ed12ME, ed12FE, ed17MC, ed17FC) was calculated and compared to the total average (x_tot_) ion intensity for each peptide (x_class_-x_tot_). A PCA model with two principal components (C1-C2) explained ~42% of the variation in the data (R2X = 0.421; Q2X = 0.298), Eigen values for the components being 11 (C1), 3.33 (C2) (Additional file 1). No patterning was discovered for sex, leaving age and EE_2 _(corresponding to four classes in the data; combined male and female ed12C, ed12E, ed17C and ed17E) as parameters for further supervised multivariate analysis. An optimized class separation based on the two variables of age and EE_2 _(corresponding to four classes: ed12C, ed12E, ed17C and ed17E) exposure was conducted by means of Partial Least Square Discriminant Analysis (PLS/DA). The age & EE_2 _PLS/DA resulted in a model with three components (DC1-3; Eigen values 11, 2.62 and 2.12) explaining ~72% of the variation in the data (R2X = 0.464; Q2X = 0.367 and R2Y = 0.722, Additional file 2). See also additional file 3 for additional information. The most influential peptides for the separation of the four classes were identified by calculating the VIP (variable importance in the projection). Peptides with large VIP, larger than 1, are the most relevant for explaining the separation. Confidence intervals for VIP were derived from jack knifing. Both PCA and PLS/DA models were optimized using Q2 cross validation scores and had the Hotelling's T^2 ^tolerance region set at 95% and the DmodX critical distance (Dcrit) set to 0.05. The median log2 ion intensity value for each peptide from the six technical replicates (Q52a-Q52f) was used for both PCA and PLS/DA analysis.

**Table 4:** Experimental groups and PLS/DA classes for age and EE2 exposure

Experimental groups	No Individuals^1^	PLS/DA classes^2^
ed12 male controls (ed12MC)	5	ed12C
ed12 female controls (ed12FC)	4	
ed12 male EE2 (ed12ME)	4	ed12E
ed12 female EE2 (ed12FE)	4	
ed17 male controls (ed17MC)	4	ed17C
ed17 female controls (ed17FC)	4	
ed17 male EE2 (ed17ME)	4	ed17E
ed17 female EE2 (ed17FE)	5	

^1. ^Not including technical replicates, and outliers Q62, Q10 and Q30

^2. ^Partial Least Square Discriminant Analysis

To test for differences in peptide expression between groups (not including outlier samples Q10, Q30 and Q62) and using the median value of the six technical replicates (Q52a-Q52f)) a linear model was employed estimating the effects of the fixed effects age, sex and treatment. The linear model used was:

where *μ *was the average peptide expression, *A*_*i *_represented the age factor (*i *= 12,17), *S*_*j *_the sex factor (*j *= M, F), *T*_*k *_the treatment factor (*k *= C, T) and *AS*_*ij*_, *AT*_*ik*_, *ST*_*jk *_and *AST*_*ijk *_the interaction factors and ε_*ijkl *_was the NID(0, σ^2^) error component. Marginal sum of squares were calculated for main effects and interaction effects. An F-test was performed to test the significance of the model and the p-values were adjusted for multiple testing [50]. The joint significance of all interaction terms was tested using Wald F-test. Least squares means for statistical significant (p < 0.05) main effects were calculated (see additional file 4). For pair wise t-testing, three levels of statistical significance were used: p < 0.05 (*), p < 0.01 (**) and p < 0.001 (***) (see Additional file 5). The *nlme *[51] library available in the R [52] software was used for the statistical analysis. Follow up comparative sequence analysis for specific peptides was done using BLAST 2 sequences alignment and ClustalW.

## Abbreviations

C1: Principal component 1; C2: Principal component 2; CRMP-2: Collapsin response mediator protein-2; CTFP: C-terminal flanking peptide; DA: discriminant analysis (variable); DC1: PLS/DA component 1; DC2: PLS/DA component 2; DC3: PLS/DA component 3; ed: embryonic day; ed12: embryonic day 12; ed17: embryonic day 17; ed12C: embryonic day 12 controls, males and females; ed12E: embryonic day 12 ethinylestradiol exposed, males and females; ed17C: embryonic day 17 controls, males and females; ed17E: embryonic day 17 ethinylestradiol exposed, males and females; ed12MC: embryonic day 12 male controls; ed12ME: embryonic day 12 male ethinylestradiol exposed; ed12FC: embryonic day 12 female controls; ed12FE: embryonic day 12 female ethinylestradiol exposed; ed17MC: embryonic day 17 male controls; ed17ME: embryonic day 17 male ethinylestradiol exposed; ed17FC: embryonic day 17 female controls; ed17FE: embryonic day 17 female ethinylestradiol exposed; EE_2_: Ethinylestradiol; FTICR-MS: Fourier transform ion cyclotron resonance mass spectrometer; GnIH: Gonadotropin inhibitory hormone; GnIH-RP2: Gonadotropin inhibitory hormone related peptide 2; GRP: Gastrin releasing peptide; HPG: hypothalamic-pituitary-gonadal; LC-MS: Liquid chromatography-mass spectrometry; LTQ-MS: Linear ion trap mass spectrometer; MERF: Met-enkephalin-Arg-Phe; MERSL: Met-enkephalin-Arg-Ser-Leu; NKA: Neurokinin A; NPK: Neuropeptide K; PCA: Principal component analysis; PENK: Preproenkephalin A; PLS/DA: Partial Least Square Discriminant Analysis; PNOC: Prepronociceptin; PPT: Preprotachykinin; SCG1: Secretogranin 1; SCG2: Secretogranin 2; SCG5: Secretogranin 5; SP: Substance P; VIP: Variable importance in the projection

## Authors' contributions

BS, KK and AM designed the experiments. AM performed the experiment related to the tissue sampling. BS and HA prepared the samples for mass spectrometry analysis, assisted by KS and AN. BS, MF and MMS interpreted the mass spectra, MF created the peptide databases and MMS identified the posttranslational modifications. BS and KK analyzed the expression data. Denator AB, BB, PEA and LD contributed funding, reagents, materials, and analysis tools. BS drafted the manuscript with contributions from all authors. All authors read and approved the final manuscript.

## Supplementary Material

Additional file 1 **PCA images**. PCA of peptide data with associated Eigen values, and SIMCA Q2 prediction values (cumulative and component specific). Shows how additional principal components relate to the first two in predictive power. Click here for file

Additional file 2 **PLS/DA goodness of prediction (Q2X) plot for age and EE_2 _effects**. PLS/DA data including Eigen values, and SIMCA Q2 prediction values (cumulative and component specific). Shows how additional principal components relate to the first three in predictive power. Click here for file

Additional file 3 **Ed12 PCA, PLS/DA and Coomans' plots for EE_2 _effects**. Data on how EE2 exposure influences the separation of samples at ed12 and ed17. A, Coomans' plots based on ed12 and ed17 PCA models indicate that ed12 is associated with a more distinct EE2 effect. B, and C, PLS/DA models of ed12 and ed17 also indicate that ed12 is more distinct in its EE2 effects and that the top candidate peptide influencing ed12 EE2 effects is NPK. Click here for file

Additional file 4 **Preproenkephalin and GnIH-RP2 peptides**. Alignment of identified Japanese quail peptide sequences to precursor protein sequences for Preproenkephalin and GnIH and annotation of likely neuropeptide cleavage sites. Click here for file

Additional file 5 **F-statistics**. Table with statistic data values. Click here for file
