# Supplementary figures and images for: Neuropeptidomic analysis of the embryonic Japanese quail diencephalon
Source: BMC Dev Biol. 2010 Mar 18;10:30. doi: 10.1186/1471-213X-10-30 (PMC2851587; doi:10.1186/1471-213X-10-30)

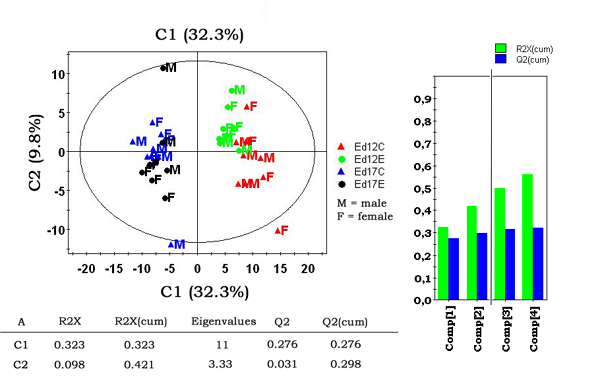

Supplement: Additional file 1 — PCA images. PCA of peptide data with associated Eigen values, and SIMCA Q2 prediction values (cumulative and component specific). Shows how additional principal components relate to the first two in predictive power. [file 1471-213X-10-30-S1.TIFF]

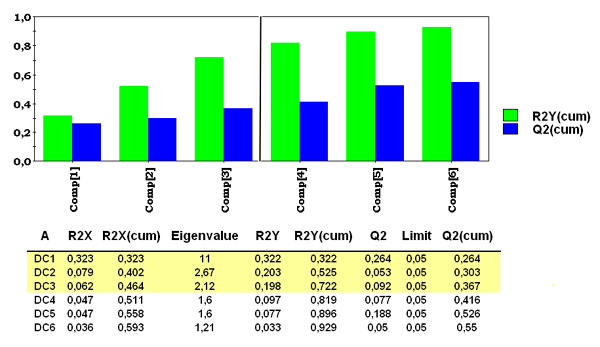

Supplement: Additional file 2 — PLS/DA goodness of prediction (Q2X) plot for age and EE2 effects. PLS/DA data including Eigen values, and SIMCA Q2 prediction values (cumulative and component specific). Shows how additional principal components relate to the first three in predictive power. [file 1471-213X-10-30-S2.TIFF]

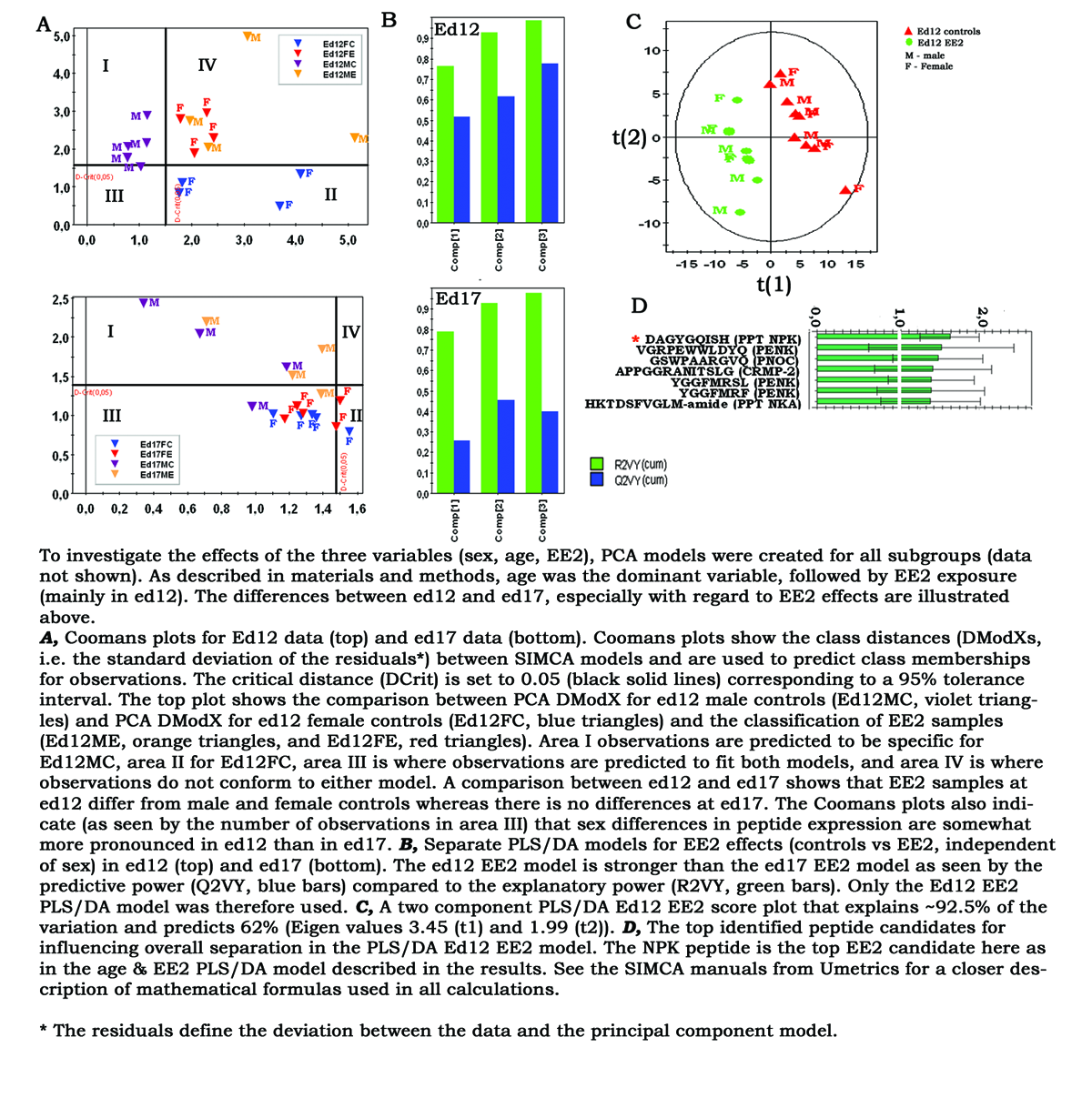

Supplement: Additional file 3 — Ed12 PCA, PLS/DA and Coomans' plots for EE2 effects. Data on how EE2 exposure influences the separation of samples at ed12 and ed17. A, Coomans' plots based on ed12 and ed17 PCA models indicate that ed12 is associated with a more distinct EE2 effect. B, and C, PLS/DA models of ed12 and ed17 also indicate that ed12 is more distinct in its EE2 effects and that the top candidate peptide influencing ed12 EE2 effects is NPK. [file 1471-213X-10-30-S3.TIFF]

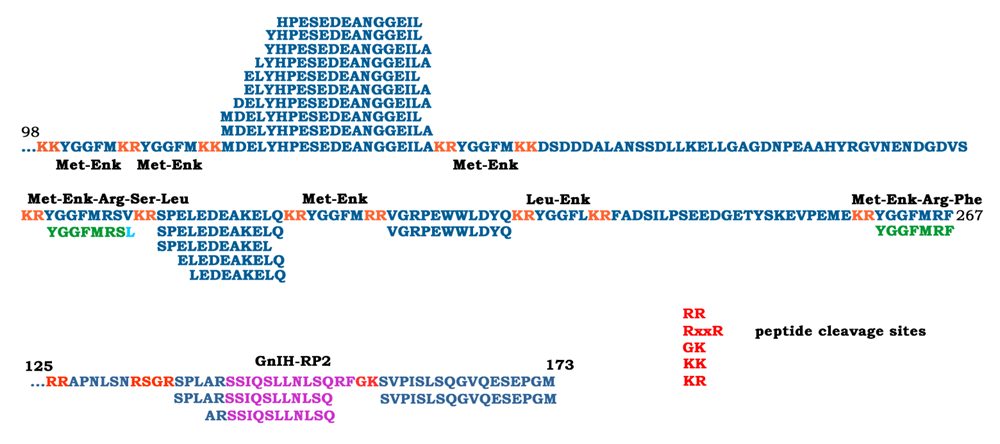

Supplement: Additional file 4 — Preproenkephalin and GnIH-RP2 peptides. Alignment of identified Japanese quail peptide sequences to precursor protein sequences for Preproenkephalin and GnIH and annotation of likely neuropeptide cleavage sites. [file 1471-213X-10-30-S4.TIFF]
